# The hereditary nature of small cell carcinoma of the ovary, hypercalcemic type: two new familial cases

**DOI:** 10.1007/s10689-016-9957-6

**Published:** 2016-11-19

**Authors:** Leora Witkowski, Nancy Donini, Rebecca Byler-Dann, James A. Knost, Steffen Albrecht, Andrew Berchuck, W. Glenn McCluggage, Martin Hasselblatt, William D. Foulkes

**Affiliations:** 1 Department of Human Genetics, McGill University, Montreal, QC Canada; 2 Lady Davis Institute and Segal Cancer Center, Jewish General Hospital, 3755 Cote Ste Catherine Road, Montreal, QC H3T 1E2 Canada; 3 College of Medicine at Peoria, University of Illinois, Peoria, IL USA; 4 Illinois Cancer Care, Peoria, IL USA; 5 Department of Pathology, McGill University, Montreal, QC Canada; 6 Duke University Medical Center, Durham, NC USA; 7 Department of Pathology, Belfast Health and Social Care Trust, Belfast, UK; 8 Institute of Neuropathology, University Hospital Münster, Münster, Germany; 9 Department of Medical Genetics and Cancer Research Program, Research Institute, McGill University Health Centre, Montreal, QC Canada

**Keywords:** Ovarian cancer, Hereditary, SCCOHT, SMARCA4, Rhabdoid, Mutation

## Abstract

Small cell carcinoma of the ovary, hypercalcemic type, (SCCOHT) is the most common undifferentiated ovarian cancer in women aged under 40 years. SCCOHT is a monogenic disease, characterized by germline and somatic *SMARCA4* mutations. Recent studies have stressed its morphological and clinical similarity to malignant rhabdoid tumours, which are usually caused by mutations in the related gene, *SMARCB1*. While familial tumours are rare, the incidence of germline mutations is relatively high, with up to 43% of SCCOHTs and 35% of rhabdoid tumours caused by germline mutations in *SMARCA4* and *SMARCB1,* respectively. We report two new familial cases of SCCOHT. Affected members in both families and the associated tumours were found to carry *SMARCA4* germline and somatic mutations, respectively, leading to loss of SMARCA4 protein expression in the tumours. Despite the rarity of familial SCCOHT, the high incidence of germline mutations is important to note, as without a family history of the disease, the hereditary nature of SCCOHT may be missed, especially if the mutation was inherited from the father or acquired de novo. The similarity between SCCOHT and rhabdoid tumours should be recognized, as infant carriers of *SMARCA4* mutations may be at risk for these tumours in addition to SCCOHT.

## Introduction

Small cell carcinoma of the ovary, hypercalcemic type (SCCOHT) is the most common undifferentiated form of ovarian cancer in women below age 40. It is an aggressive cancer with 5-year survival rates of 53.8% in stage I disease [1]. SCCOHT is a monogenic and at times hereditary disease, characterized by germline and somatic mutations in the chromatin remodelling gene, *SMARCA4*, a member of the SWI/SNF complex [1]. Further analyses have revealed that these tumours are very similar to rhabdoid tumours (RTs) on clinical, morphological, and genetic levels. As such, we have proposed that SCCOHT should be regarded as an RT of the ovary [2]. RTs are pediatric soft tissue tumours that usually arise in the kidney but can arise elsewhere, and along with the intracranial variant, called atypical teratoid/rhabdoid tumours (ATRTs), they are usually characterized by mutations in *SMARCB1*, another gene in the SWI/SNF complex. In rare instances, however, RTs are caused by mutations in *SMARCA4* [3]. These mutations almost always lead to loss of their encoded protein (SMARCA4 or SMARCB1) by immunohistochemistry, making this an important diagnostic marker for these tumours [1].

In rare instances, RTs and SCCOHTs have been found to arise in familial settings due to germline mutations in either *SMARCA4* or *SMARCB1* [1]. Up to 35% of RTs are caused by germline *SMARCB1* mutations [4], and up to 43% of SCCOHTs by germline *SMARCA4* mutations [1]; this has implications for management of the diseases in the affected families. Germline *SMARCB1* or *SMARCA4* mutations define rhabdoid tumour predisposition syndrome type 1 (RTPS1; OMIM #609322) or type 2 (RTPS2, OMIM #613325), respectively. Although the ovarian type of RT (SCCOHT) is almost always caused by mutations in *SMARCA4*, and extra-ovarian RT by mutations in *SMARCB1*, it is possible for multiple tumour types within the rhabdoid tumour spectrum to be caused by the same mutation [3–5].

Here we describe two previously unpublished families, both consisting of two females with SCCOHT. The affected women were diagnosed between the ages of 23 and 36, and three of the four eventually succumbed to their disease. We discuss the implications of familial occurrence of SCCOHT and propose necessary steps for diagnosis and management of the disease in a genetic context.

## Materials and methods

### Samples

DNA was extracted from patients’ blood and FFPE tumour tissue as previously described. RNA was extracted from patients’ blood as previously described [6].

Sanger sequencing and immunohistochemistry were performed as previously described [5].

## Results

### Family 1

**Fig. 1:**
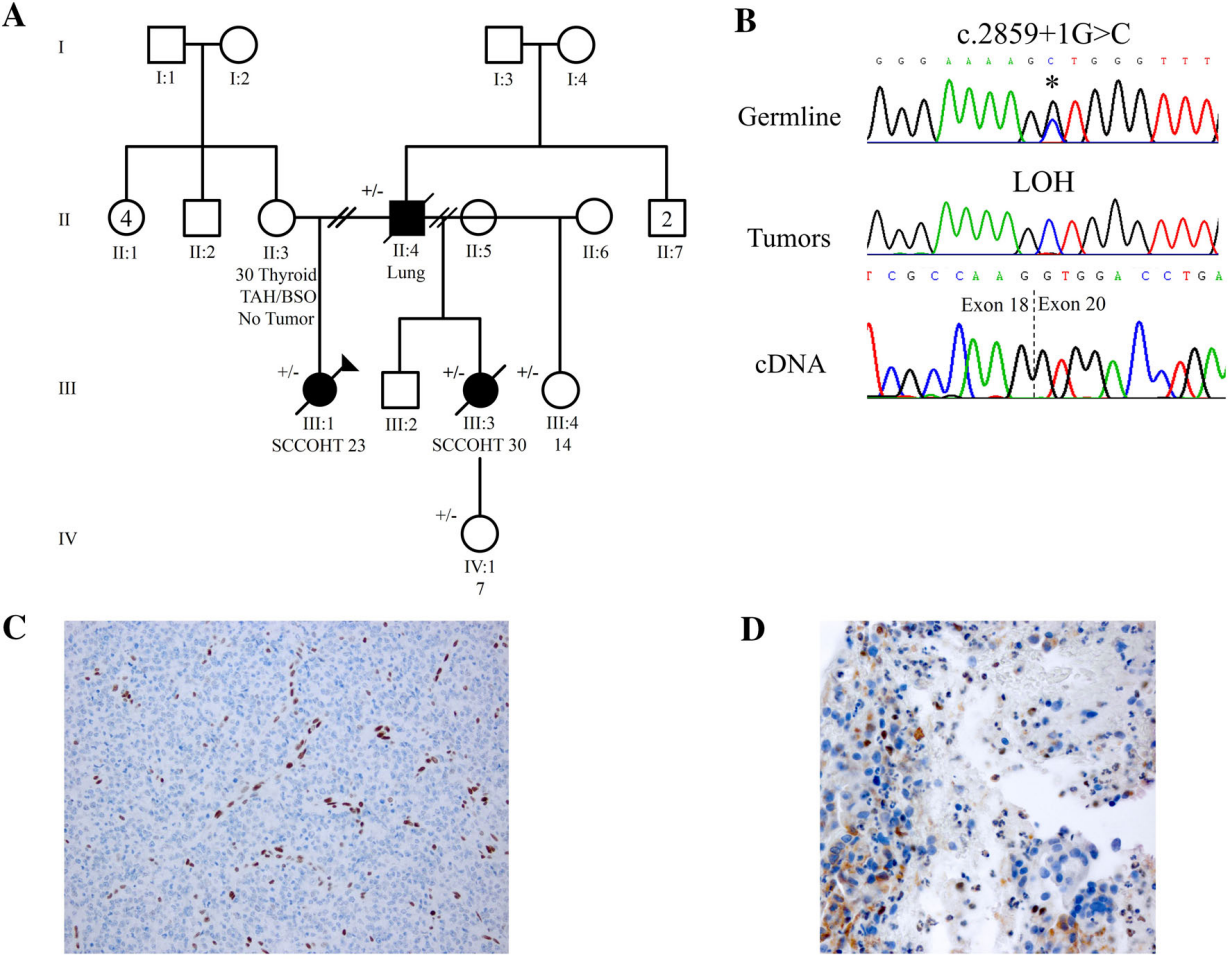
Family 1. **a** Pedigree of family 1. **b** Mutations found by Sanger sequencing in affected patients. *Top*: Germline mutation found in both patients; *middle*: representative chromatogram from patient III:1 showing somatic LOH found in tumours of both patients; *bottom*: cDNA sequencing across mutation, showing that splice mutation leads to skipping of exon 19. Loss of expression was seen in both SCCOHT tumors and representative SMARCA4 immunohistochemistry is shown in two tumors—**c** in the SCCOHT from patient III:3, with complete loss of SMARCA4 staining and positive internal controls and **d**: in the lung tumor from patient II:4. In **d** immunohistochemistry for SMARCA4 shows loss of nuclear SMARCA4 staining in pleomorphic tumor cells, but retained staining in small round lymphocytic nuclei (internal positive control). Original magnification 600×. TAH/BSO, total abdominal hysterectomy/bilateral salpingo-oophorectomy

Family 1 consists of a proband who was diagnosed with SCCOHT at age 23 years (Fig. 1a, patient III:1) and her paternal half-sister (Fig. 1a, patient III:3), who was diagnosed with SCCOHT at age 30. Patient III:1 was diagnosed with FIGO stage IC SCCOHT of the right ovary. Bilateral salpingo-oophorectomy and omentectomy, followed by cisplatin and etoposide with adjuvant pelvic radiation achieved a complete response. However, she relapsed in a periaortic lymph node eighteen months after diagnosis and received further cisplatin and etoposide as well as adjuvant radiotherapy to the periaortic nodal recurrence. She underwent subsequent exploratory laparotomy and resection of the right periaortic mass. She is currently recovering from surgery.

Patient III:3, the half-sister of patient III:1, was diagnosed with FIGO stage IIIB SCCOHT of the left ovary. She underwent a left salpingo-oophorectomy, right oophorectomy, omentectomy, and peritoneal biopsies. Her omentum and peritoneal biopsies showed involvement by tumour. She received cisplatin plus etoposide for one cycle with no response. Six weeks after diagnosis she received palliative radiation for three weeks and died 2 weeks later, three months after the original diagnosis.

The patients’ family history is remarkable in that their father (Fig. 1a, patient II:2), a smoker, had died from metastatic lung carcinoma at age 53 with underlying chronic obstructive pulmonary disease. Histologically, his lung tumor was a mucinous adenocarcinoma without any rhabdoid features. All three patients were found to carry a germline splice mutation in the *SMARCA4*: c.2859+1G>C, which led to an in-frame deletion of exon 19 (Fig. 1b). Both women showed loss of heterozygosity (LOH) of the mutation in their SCCOHT (Fig. 1b). The SMARCA4 protein was immunohistochemically lost in both SCCOHTs (Fig. 1c) and the lung carcinoma (Fig. 1d). The second half-sister of the proband (Fig. 1a, patient III:4) as well as the daughter of patient III:2 (Fig. 1a, patient IV:1) were both found to carry the familial germline mutation. Patient III:4 underwent a prophylactic oophorectomy, while patient IV:1 is being followed with biannual pelvic ultrasonography. It should be noted that this screening has not been proven to be effective and should not replace oophorectomy as a management strategy, particularly for those women who have completed child-bearing.

### Family 2

**Fig. 2:**
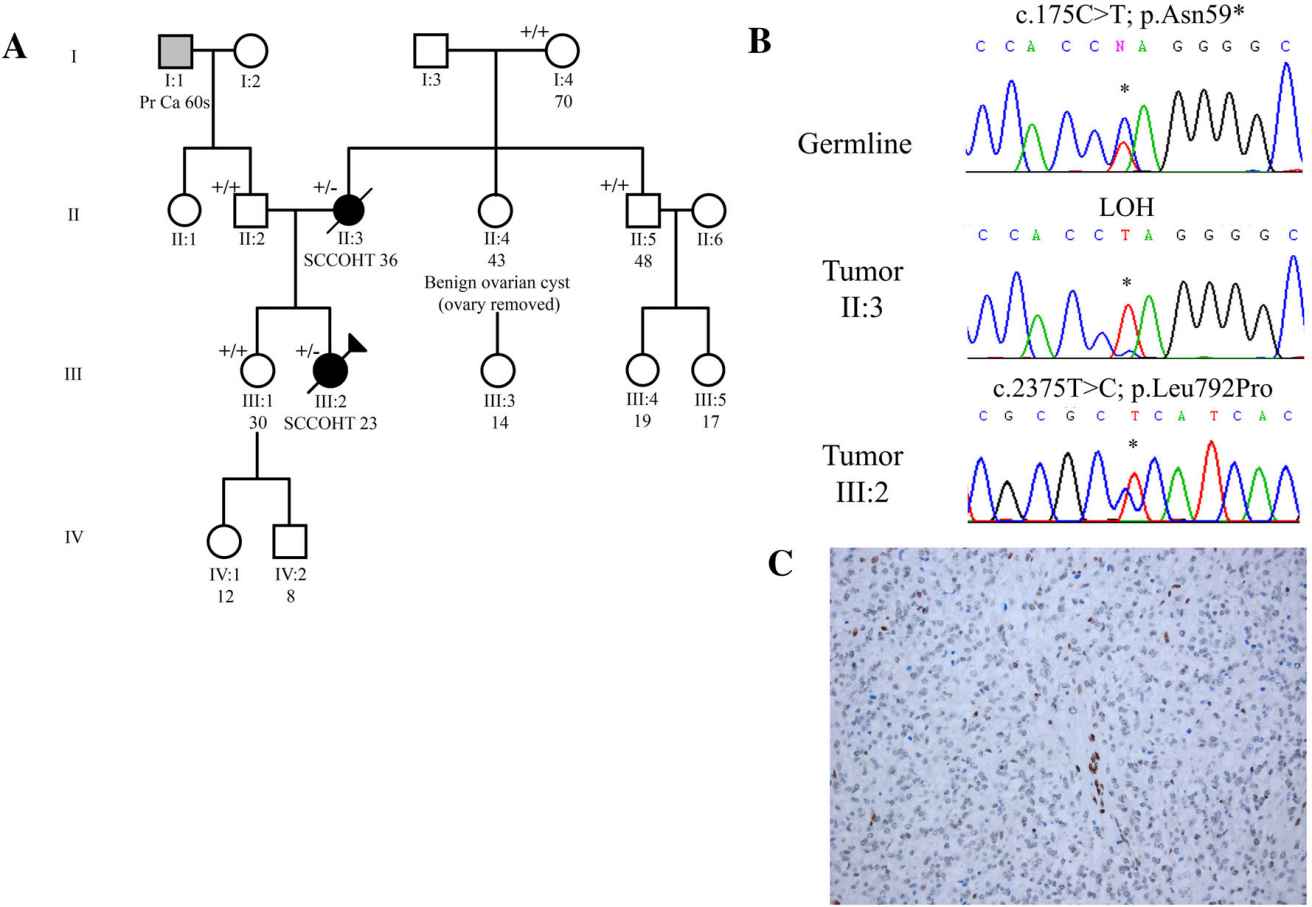
Family 2. **a** Pedigree of family 2. **b** Mutations found by Sanger sequencing in affected patients. *Top* germline mutation found in both patients; *middle* LOH seen in tumour from patient II:3; *bottom* somatic LOH found in tumours of both patients. **c** Representative SMARCA4 immunohistochemistry in tumour of patient III:2. Loss of expression was seen in both patients. Pr Ca, Prostate cancer

Family 2 consists of a proband who was diagnosed with SCCOHT at age 23 (Fig. 2, patient III:2) and her mother (Fig. 2, patient II:3), who had been diagnosed with an ovarian “rhabdoid tumour”, consistent with a diagnosis of SCCOHT 19 years earlier, at age 36 (see below). Patient III:2 (Fig. 2) was diagnosed with FIGO stage IIIB SCCOHT of the right ovary. She underwent a right salpingo-oophorectomy, an omentectomy, and resection of right periaortic and pelvic lymph nodes. There was tumour involvement of the abdominal peritoneum and metastases to her pelvic and periaortic lymph nodes. She received 6 cycles of chemotherapy, consisting of cisplatin and etoposide. She then received radiotherapy and single agent taxol for 4 cycles but rapidly progressed and died 14 months post diagnosis.

Patient II:3 was the mother of Patient III:2. At age 36, she was diagnosed with a “poorly differentiated adenocarcinoma with extensive rhabdoid features” of her right ovary. We now know that this description is compatible with a diagnosis of SCCOHT, but when she was diagnosed, SCCOHT was a relatively newly described entity (first described in 1979 [7]). The patient underwent a total abdominal hysterectomy, bilateral salpingo-oophorectomy, omentectomy, and tumour debulking. Due to her unstable condition post-operatively no chemotherapy was given. The patient died three weeks after her original diagnosis.

Aside from the two affected women, the family history was unremarkable (Fig. 2a). The *SMARCA4* gene was sequenced in germline and tumour DNA of both patients and a germline nonsense mutation was found: c.175C>T; p.Asn59* (Fig. 2b). The tumour of patient III:2 harboured a second somatic mutation: c.2375T > C; p.Leu792Pro. The tumours of both patients displayed loss of the SMARCA4 protein by immunohistochemistry (Fig. 2C). The sister of the proband did not carry the familial germline mutation.

## Discussion

Here we report two new familial occurrences of SCCOHT. Previously, only four familial cases of SCCOHT were sequenced, and all affected patients were found to carry *SMARCA4* mutations with second somatic mutations in their tumours (Table 1) [1]. While the incidence of SCCOHT occurring in families is low, the incidence of germline mutations in SCCOHT patients is relatively high (43%) [1]. This is likely due to the fact that this tumour characteristically occurs in young females (and at a younger age in those with germline mutations than those with only somatic mutations [1]). Most carriers are diagnosed prior to having children and either die of their disease, or survive infertile due to therapy. Furthermore, half of patients with germline mutations have been found to inherit the mutation from their father [1]. Only one case of RT of the ovary has been reported to be due to a de novo germline mutation, and the daughter of the patient developed an ATRT [5].

The unknown penetrance of these mutations remains a challenge when counselling patients and their families. Only one female *SMARCA4* carrier has been reported to remain healthy past her sixth decade; this was the grandmother of an ATRT patient [3]. Further testing of affected and unaffected family members will hopefully elucidate the true penetrance and allow carriers to be more informed when making potentially life-altering decisions, such as prophylactic oophorectomies [8].

Due to the high incidence of germline mutations in SCCOHT, it is recommended that all patients with the disease undergo genetic testing. Although it has not been shown to alter the treatment or outcome of patients, it can benefit relatives who may carry the mutation. Female carriers of truncating mutations are at risk for SCCOHT, and infant carriers of both genders may be at risk for RTs. *SMARCA4*-mutated RTs have not been seen in patients older than 46 months [3], so the development of these tumours in older carriers is unlikely. However, the oldest woman to date diagnosed definitively with SCCOHT (showing loss of SMARCA4 staining in her tumour) was 56 years old at diagnosis. As *SMARCA4* mutations overlap between SCCOHT and RTs, it is still unknown why patients develop one tumour over the other. While the lung tumour of the father in Family 1 showed loss of expression of SMARCA4, it is unclear whether the cancer was related to the SMARCA4 germline mutation; the patient was a smoker and many lung tumours display loss of SMARCA4 expression [9].

The types of *SMARCA4* mutations seen in SCCOHT vary, yet all but two have led to loss of expression of the protein, with the remaining two being a missense and an in-frame deletion [1]. Germline mutations in *SMARCA4*, *SMARCB1*, and other SWI/SNF components also cause Coffin-Siris syndrome (CSS) [10, 11], a developmental disorder primarily characterized by developmental delay and intellectual and physical disabilities. Interestingly, germline *SMARCA4* and *SMARCB1* mutations causing CSS have mostly been missense and de novo, whereas those causing RTs or SCCOHT have mostly been truncating and inherited, with only one reported RT caused by a de novo *SMARCA4* mutation [5]. Unlike in SCCOHT and RTs, no overlap of mutations has been reported between CSS and either SCCOHT or RT. Furthermore, no patients with SMARCA4-deficient cancers have been reported to show a CSS phenotype, and no CSS patients have been found to develop RTs. However, one patient with a *SMARCB1* mutation and CSS has been reported to develop schwannomatosis, another type of tumour caused by germline *SMARCB1* mutations [12]. The mutations leading to schwannomatosis are most often missense variants, but may be loss of function as well. Interestingly, in some patients with loss of function mutations, the *SMARCB1* mRNA has been found to escape degradation by reinitiating translation at the AUG codon encoding methionine at position 27 of the SMARCB1 protein [13]. While it is still not entirely clear why some mutations in the SWI/SNF complex predispose to cancer, while others lead to intellectual disability, but it has been postulated that the mutations in any of the SWI/SNF complex members that lead to developmental disorders exert either dominant-negative or gain-of-function effects, while those leading to SCCOHT are loss-of-function mutations [10]. Similarly, both loss of function and missense mutations in *SMARCB1* can lead to schwannomatosis [14], and it still remains unclear why some carriers develop schwannomas, while other develop RTs.

Although the familial incidence of SCCOHT is low, it is important to note the high fraction caused by germline *SMARCA4* mutations and to recognize that even without a family history, it may be hereditary, for example if the patient has inherited a germline mutation from her father or acquired one de novo. Furthermore, the similarity between SCCOHT and RTs is striking and, in addition to SCCOHT, infant *SMARCA4* mutation carriers may be at risk for these tumours.
